# Liraglutide Exerts Antidiabetic Effect via PTP1B and PI3K/Akt2 Signaling Pathway in Skeletal Muscle of KKAy Mice

**DOI:** 10.1155/2014/312452

**Published:** 2014-08-11

**Authors:** Wenjun Ji, Xinlin Chen, Juan Lv, Meng Wang, Shuting Ren, Bingxiang Yuan, Bing Wang, Lina Chen

**Affiliations:** ^1^College of Medicine, Xi'an Jiaotong University, Xi'an, Shaanxi 710061, China; ^2^Xi'an No. 1 Hospital, Xi'an, Shaanxi 710002, China

## Abstract

*Background*. Liraglutide (a glucagon-like peptide 1 analog) was used for the treatment of type 2 diabetes (T2DM) which could produce glucose-dependent insulin secretion. *Aim*. The aim was to investigate whether liraglutide could improve myofibril and mitochondria injury in skeletal muscle and the mechanisms in diabetic KKAy mice. *Method*. We divided the male KKAy mice into 2 groups: liraglutide group (250 *μ*g/kg/day liraglutide subcutaneous injection) and model group; meanwhile, the male C57BL/6J mice were considered as the control. After 6 weeks, the ultrastructure of skeletal muscle was observed by electron microscope. The gene expressions of protein tyrosine phosphatase 1B (PTP1B), phosphatidylinositol 3-kinase (PI3K), and glucose transporter type 4 (GLUT4) were determined by real-time PCR. The protein levels of the above molecules and phospho-Akt2 (p-Akt2) were measured by Western blot. *Results*. Liraglutide significantly ameliorated the injury of mitochondria by increasing the number (+441%) and the area (+113%) of mitochondria and mitochondrial area/100 *µ*m^2^ (+396%) in skeletal muscle of KKAy mice. The results of real-time PCR and Western blot showed that liraglutide downregulated PTP1B while it upregulated PI3K and GLUT4 (*P* < 0.01). The protein level of p-Akt2/Akt2 was also increased (*P* < 0.01). *Conclusion*. These results revealed that liraglutide could improve myofibril and mitochondria injury in skeletal muscle against T2DM via PTP1B and PI3K/Akt2 signaling pathway.

## 1. Introduction

Until 2012, the morbidity of diabetes in the world had reached 6.4%, which seriously affected our normal life [1] and 80%–90% of the diabetes was type 2 diabetes mellitus (T2DM). Furthermore, diabetes with sustained elevated blood glucose would destroy multiple organs; it may cause diabetic foot [2], diabetic nephropathy, cerebrovascular disease, heart disease, skin disease, and so on. So it had no time to delay for us to overcome the diabetes, especially T2DM. T2DM was characterized by insulin resistance and regression of *β*-cell [3].

In recent years, glucagon-like peptide 1 (GLP-1), which was secreted from intestinal L cells, had been reported to decrease blood glucose [4, 5], increase insulin secretion, and improve the damage of *β*-cell [6]. Liraglutide as a kind of GLP-1 analogue expressed 97% homoousia for amino acid sequences with GLP-1 of human [7]. And it had been applied to the treatment of T2DM since 2009. It had been reported that liraglutide could dose-independently enhance glucose-dependent insulin secretion without causing hypoglycemia, which indicated an insulin-like effect on T2DM, decreased postprandial glucose levels [8], improved glycemic control, and lowered insulin dose [9] in patients with T2DM. Protein tyrosine phosphatase 1B (PTP1B) was a novel target for T2DM and had been proved to play a vital role in the negative regulation of insulin signal transduction [10]. Phosphatidylinositol 3-kinase (PI3K), protein kinase B-2 (Akt2), and glucose transporter type 4 (GLUT4) played an important role in insulin transduction. PI3K was the regulatory molecule and the function was to activate downstream molecules. The expression of GLUT4 and phosphorylation of Akt2 were quite associated with T2DM [11, 12]. All of those signal molecules mentioned above were confirmed to be closely related to insulin transduction [13]. However, whether liraglutide produces antidiabetic effects through these signal pathways remained unclear.

We had ever reported the effect of liraglutide on blood glucose levels with oral glucose tolerance test (OGTT) and insulin tolerance test (ITT) and glycogen, which showed that liraglutide could improve glucose metabolism and insulin resistance [14]. More rapid increase and persistent decrease of blood glucose level in liraglutide and control groups were observed and the area under the curve (AUC) of OGTT was significantly lower than KKAy mice. In addition, fasting blood glucose (FBG) of ITT in control and liraglutide groups was persistently decreased after 40 min since insulin injection, whereas glucose levels in KKAy mice were elevated. Meanwhile, the AUC of ITT in liraglutide and control groups had a significant decrease compared to KKAy mice. The level of skeletal muscle glycogen was significantly lower in diabetic model mice, compared with normal mice; however, liraglutide treated diabetic mice had greatly increased skeletal muscle glycogen levels, similar to the normal group. It was reported that diabetes would cause kidney [15], skeletal muscle [16], and liver damage [17], and mitochondria injury [18]. Therefore, we investigated whether liraglutide could improve tissue damage and mitochondria injury. In this study, we observed the ultrastructure and micrographs of mitochondria and myofibril and aimed to determine whether liraglutide could improve myofibril damage and mitochondria injury and the relationship with the PI3K/Akt signaling pathway.

## 2. Materials and Methods 

### 2.1. Animals and Experimental Procedures

The study protocols were approved by the Ethics Committee on the Care and Management of Experimental Animals in Xi'an Jiaotong University, Xi'an, China. 11–13-week-old male KKAy mice (*n* = 12) and the same age male C57BL/6J (C57) mice (*n* = 6) were purchased from the Chinese Academy of Medical Sciences (Beijing, China). All the mice were individually housed in cages at a temperature of 20–22°C, a humidity of 45%–55% with specific pathogen-free (SPF) environment, and a 12 h light and 12 h dark cycle. All the mice drank water freely. High-fat chow with 6% fat from Beijing HFK Bioscience Company was supplied to KKAy mice and the C57 mice were given ordinary rodent diet.

After a week of acclimation, we measured the FBG levels of KKAy mice. The mice with FBG values >16.7 mmol/L were randomly divided into liraglutide group (*n* = 6, treated with 250 *μ*g/kg/day liraglutide subcutaneous injection, provided by Novo Nordisk) and model group (*n* = 6, treated with equivalent volume of normal saline). The male C57 mice (*n* = 6, treated with equivalent volume of normal saline) were considered as the control group. All the mice were treated for 6 weeks between 16:00 and 16:30 pm each day.

### 2.2. Tissue Processing

The thigh muscle tissues were divided into four specimens. One was fixed in 10% formalin for Hematoxylin-Eosin (HE) staining and one was immersed in stationary liquid for electron microscope assays. The third was immersed into TriPure RNA isolation reagent (Roche, Basel, Switzerland) and reserved at 4°C for real time-PCR examination. The fourth was frozen in liquid nitrogen for 1 minute and then stored at −80°C for Western blot analysis.

### 2.3. Electron Microscope

For electron microscope, each muscle fraction of 1 mm × 1 mm × 1 mm was fixed in 2.5% glutaraldehyde in 0.1 mol/L cacodylate buffer and postfixed in 2% osmium tetroxide. After being dehydrated in grade ethanol, samples were embedded in spur resin. Semitic sections were stained with toluidine blue. The ultrathin sections, placed on 200 mesh copper grids, were stained with uranyl acetate and lead citrate. Then we observed the samples with a transmission electron microscope. Images were analyzed by image J 1.42q. Individual mitochondrial area, mitochondrial area/100 *μ*m^2^, and mitochondrial number/10 *μ*m^2^ were determined by analyzing ten images taken at 20000× magnification, similar to methodologies [19].

### 2.4. RNA Isolation and Real-Time PCR

Total RNA was isolated from mouse skeletal muscle using the TriPure RNA isolation reagent, and two micrograms of RNA were reverse-transcribed using the Prime Script RT Master Mix (Perfect Real Time) (TaKaRa Bio, Inc., Tokyo, Japan). Quantitative real-time PCR was performed using SYBR Premix Ex Taq II (Perfect Real Time) (TaKaRa Bio, Inc., Tokyo, Japan). PCR reactions were performed in 96-well plates in an iQ5 Real-Time PCR Detection System (Bio-Rad Laboratories, Hercules, CA).

All the primers and probes for real-time PCR were obtained from TAKARA Bio. The specific primers were as follows: PTP1B, 5′-CAC AGT ACG ACA GTT GGA GTT GGA A-3′ (forward) and 5′-CAG GCC ATG TGG TGT AGT GGA-3′ (reverse); PI3 K, 5′-GCT CCT GGA AGC CAT TGA GAA-3′ (forward) and 5′-CGT CGA TCA TCT CCA AGT CCA C-3′ (reverse); GLUT4, 5′-TCT TAT TGC AGC GCC TGA GTC-3′ (forward) and 5′-GCC AAG CAC AGC TGA GAA TAC A-3′ (reverse); GAPDH, 5′-TGT GTC CGT CGT GGA TCT GA-3′ (forward) and 5′-TTG CTG TTG AAG TCG CAG GAG-3′ (reverse). GAPDH served as endogenous control. The results were normalized to GAPDH. Efficiencies of real-time PCR for the target gene and the endogenous control were approximately equal. −ΔCT expresses the difference between number of cycles (CT) of the target genes and the endogenous control. Results were expressed as 2^−ΔΔCt^ and express the x-fold increase of gene expression compared to control group. The standard curve and data analysis were produced using Bio-Rad iQ5 software (Bio-Rad Laboratories, Hercules, CA).

### 2.5. Western Blot Analysis

100 mg skeletal muscle tissue from mice was ground manually in 1 mL Radio-Immunoprecipitation Assay (RIPA) lysate. We got the supernatant by centrifuging the samples at 12000 ×g for 20 min at 4°C and determined the protein concentration of the supernatant by bicinchoninic acid (BCA) protein assay kit. Proteins (typically 50 *μ*g/lane) were separated by 10% SDS-PAGE gel and transferred to PVDF membranes (Millipore, MA, USA). Then we blocked the membranes with 5% nonfat milk, incubated them with primary antibodies and HRP conjugated secondary antibodies, and detected bands using chemiluminescence substrate reagents (Thermo, USA). Primary antibodies for PTP1B, PI3K (p85*α*), Akt2, and GLUT4 were all purchased from Abcam (Abcam, Cambridge, UK), phospho-Akt2 (p-Akt2, Ser474) from Cell Signaling Technology (CST, Bostin, USA), and *β*-actin from Santa Cruz Biotechnology (Santa Cruz, CA, USA). *β*-actin was used to normalize the result of each sample.

### 2.6. Statistical Analysis

Values were shown as means ± standard error of the means (SEM). Significant differences between the two groups were evaluated by independent sample *t*-test and ANOVA. SPSS v13.0 software was utilized to analyze data. Image J 1.42q and GraphPad Prism 5.0 software were used to perform the figures. Parameters with values of *P* < 0.05 were considered as statistical significance.

## 3. Results

### 3.1. Liraglutide Ameliorated Myofibril Damage in Skeletal Muscle of KKAy Mice

**Figure 1:**
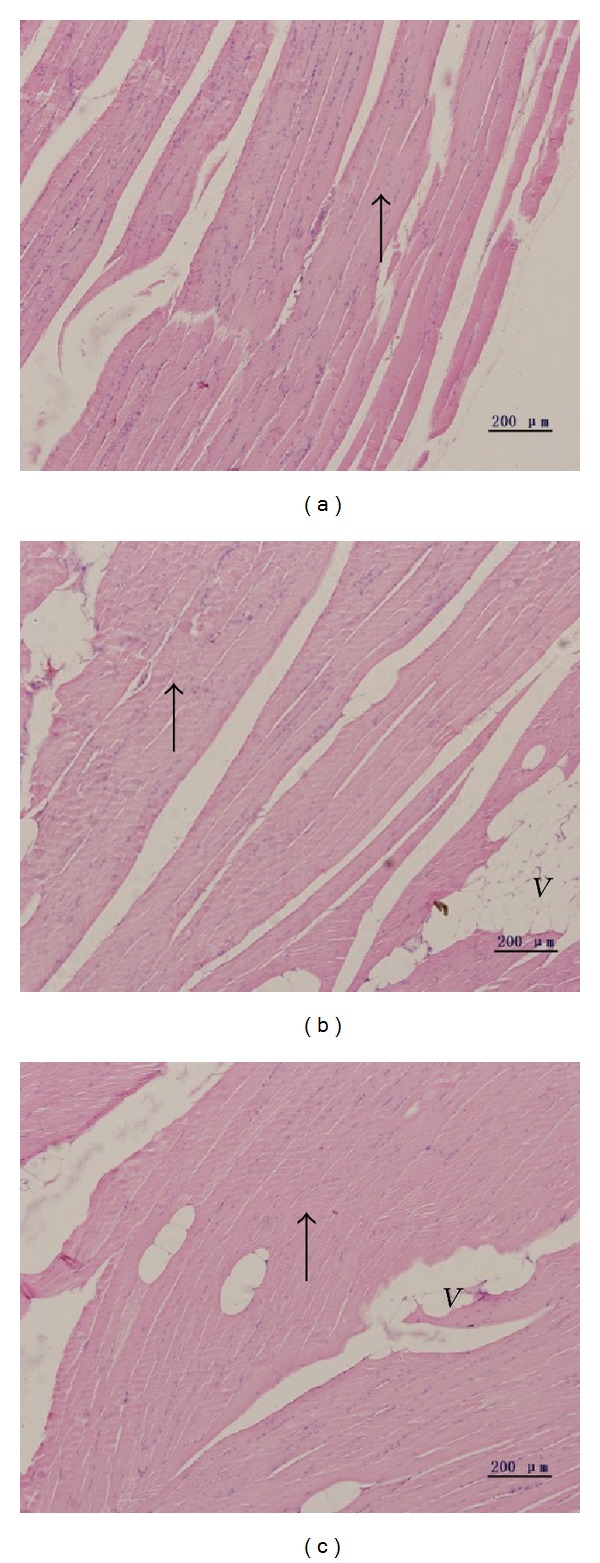
(a) Normal myofibril of skeletal muscle in normal mice, HE ×200. (b) Atrophic myofibril of skeletal muscle in diabetic model mice, HE ×200. (c) Improved myofibril of skeletal muscle after liraglutide injected for 6 weeks, HE ×200.* Black arrow*: myofibril,* V*: vacuoles.

Analysis of HE stained sections from skeletal muscle showed myofibril with clear cross striation and regular cross sections in normal C57 mice (Figure 1(a)). In KKAy mice, we observed atrophic and abnormal myofibril. Furthermore, cross striation of diabetic muscle became fuzzy and some even disappeared (Figure 1(b)). As shown in Figure 1(b), a mass of vacuoles appeared in muscle and the nucleus increased. The image of liraglutide treated diabetic mice (Figure 1(c)) showed clearer cross striation and less atrophic myofibril, compared with the diabetic mice.

### 3.2. Liraglutide Significantly Improved the Injury of Mitochondria by Increasing the Amount and the Area of Mitochondria in Skeletal Muscle of KKAy Mice

**Figure 2:**
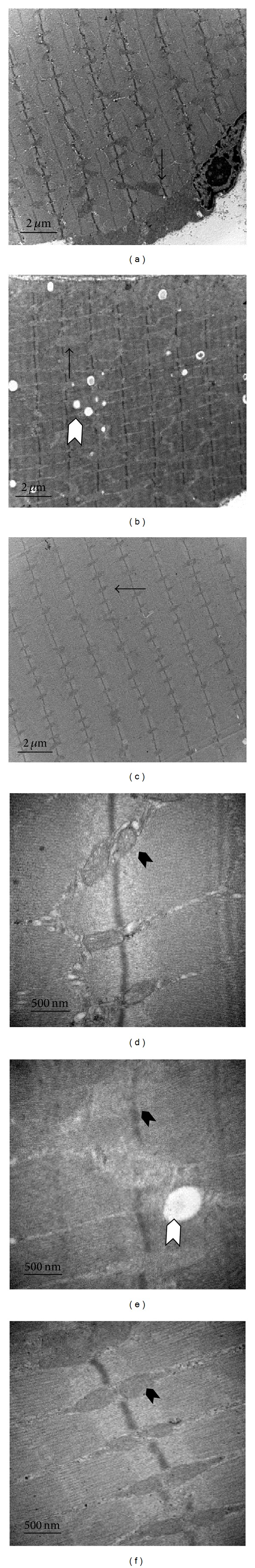
Electron micrographs of skeletal muscle tissue. Normal mitochondria and myofibril in skeletal muscle of normal mice ((a), (d)). Disordered myofibril and mitochondria with fuzzy cristae in skeletal muscle of diabetic model mice ((b), (e)). Improved myofibril and mitochondria with clearer cristae than diabetic mice in skeletal muscle of diabetic mice treated with liraglutide for 6 weeks.

**Figure 3:**
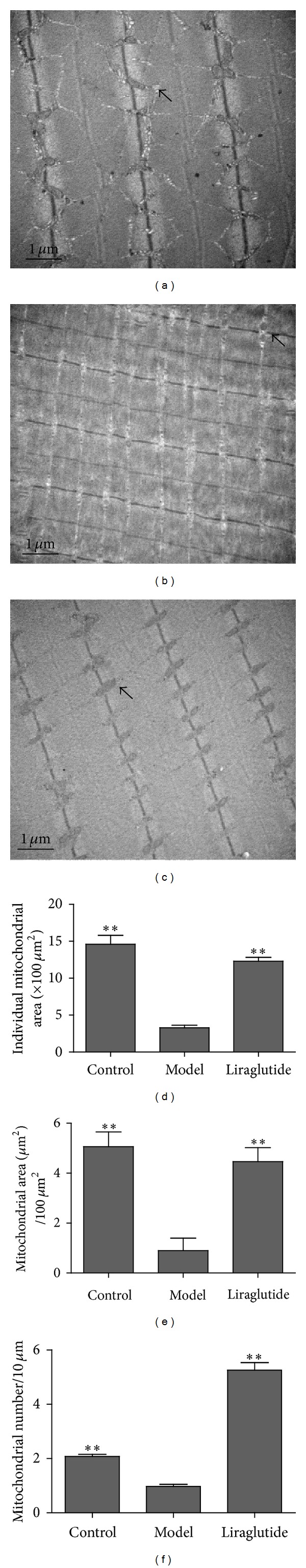
Individual mitochondrial area, mitochondrial area/100 *μ*m^2^, and mitochondrial number/10 *μ*m^2^ in normal (a), model (b), or liraglutide group (c) measured by the electron microscope. Individual mitochondrial area (d), mitochondrial area/100 *μ*m^2^ (e), and mitochondrial number/10 *μ*m^2^ (f) in skeletal muscle were significantly increased when compared with diabetic mice (data were shown as mean ± SEM, **P < 0.01 versus model group).

From the micrograph (Figure 2(a)), we observed no abnormal structural alteration in the skeletal muscle of the C57 mice. The arrangement of myofibrils and sarcomere was regular and clear. Also, density and a large number of mitochondria with clear cristae were examined in Figure 2(d). The abnormal myofibril arrangement was observed in diabetic KKAy mice. Myofibril spacing became larger and stroma increased. We could also see more lipid droplets in diabetic mice, compared with normal mice (Figure 2(b)). As shown in Figure 2(e), vacuolated, disarranged, and swollen mitochondria clusters were observed in skeletal muscle of diabetic mice. The membrane of mitochondria became fuzzy and the number declined significantly. The abnormal arrangement of myofibril was improved after 6-week treatment with liraglutide in diabetic KKAy mice (Figure 2(c)). We also found the number of lipid droplet reduced. The increased number and regular arrangement of the mitochondria in liraglutide treated diabetic mice were the biggest change we observed (Figure 2(f)). Histologically, mitochondria abnormalities in liraglutide treated mice were improved largely, compared with those with diabetic mice. Compared with diabetic KKAy mice, individual skeletal muscle mitochondrion area in liraglutide treated mice was +113% larger (*P* < 0.01) (Figure 3(d)), while mitochondrial area/100 *μ*m^2^ in liraglutide treated mice was +396% larger (*P* < 0.01) (Figure 3(e)). The mitochondrial number/10 *μ*m^2^ in liraglutide treated mice was markedly increased (+441%; *P* < 0.01) (Figure 3(f)).

### 3.3. Liraglutide Influenced Gene Expression in KKAy Mice

**Figure 4:**
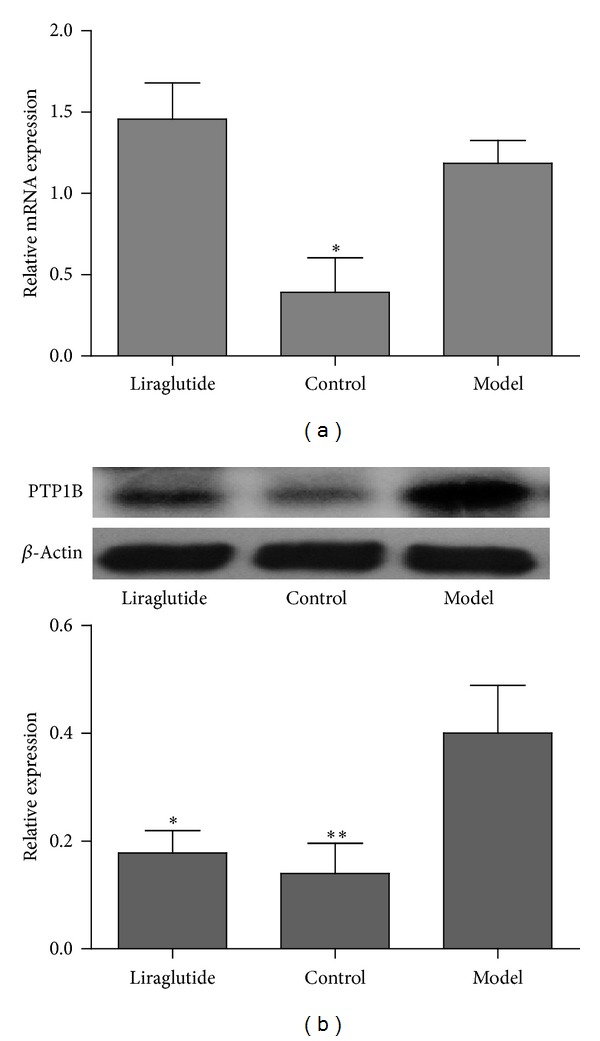
Effects of liraglutide on PTP1B expression in skeletal muscle. (a) Expression in skeletal muscle of PTP1B mRNA in normal, model, or liraglutide group. Gene expression was measured by real-time PCR. Relative PTP1B mRNA abundance was normalized by *β*-actin. Data were shown as mean ± SEM, *P < 0.05, **P < 0.01 versus model group, *n* = 5. (b) Expression of PTP1B protein in skeletal muscle from normal, model, or liraglutide group. Lysates from freshly isolated skeletal muscle tissues were measured for PTP1B expression by Western blot analysis. Results were shown as mean ± SEM, *P < 0.05, **P < 0.01 versus model group, *n* = 5.

**Figure 5:**
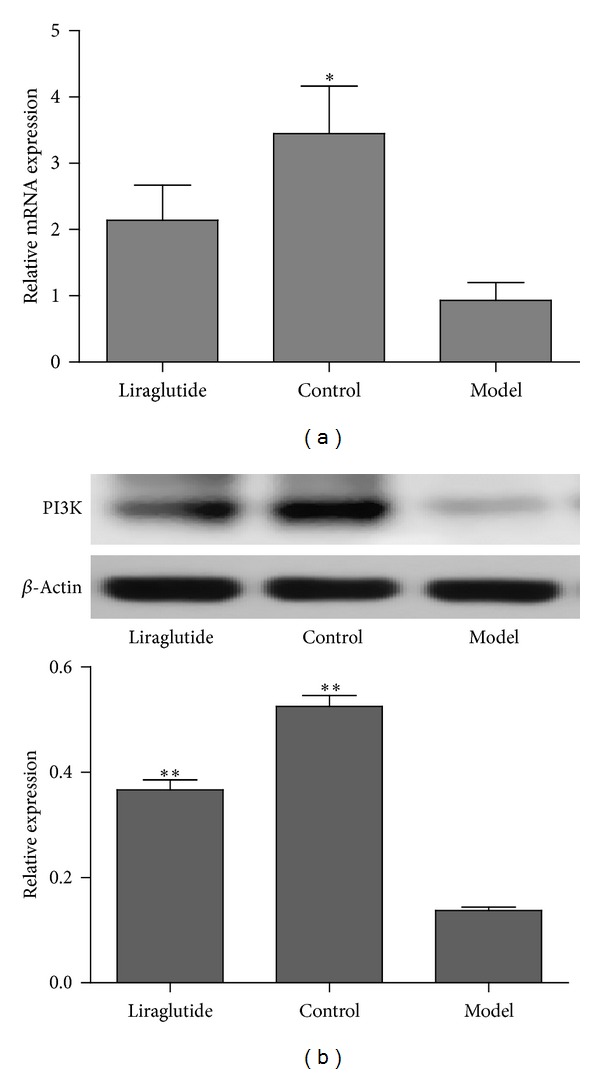
Effects of liraglutide on PI3K expression in skeletal muscle. (a) Expression in skeletal muscle of PI3K mRNA in normal, model, or liraglutide group. Gene expression was measured by real-time PCR. Relative PI3K mRNA abundance was normalized by *β*-actin. Data were shown as mean ± SEM, *P < 0.05, **P < 0.01 versus model group, *n* = 5. (b) Expression of PI3K protein in skeletal muscle from normal, model, or liraglutide group. Lysates from freshly isolated skeletal muscle tissues were measured for PI3K expression by Western blot analysis. Results were shown as mean ± SEM, *P < 0.05, **P < 0.01 versus model group, *n* = 5.

The gene expression of PTP1B in skeletal muscle was shown in Figure 4(a). Compared with the model group, the control group had a significantly lower PTP1B gene expression level (*P* < 0.05). Furthermore, we assessed the effects of liraglutide on PI3K and GLUT4 gene expression in model group. PI3K (Figure 5(a)) and GLUT4 (Figure 7(a)) gene expression was significantly increased in the control group compared with the diabetic model mice (*P* < 0.05). Although the level of GLUT4 and PI3K in liraglutide group had no significant increase, the expression was higher than model group.

### 3.4. Liraglutide Downregulated the Level of PTP1B and Regulated PI3K/Akt Signaling Pathway in Skeletal Muscle

**Figure 6:**
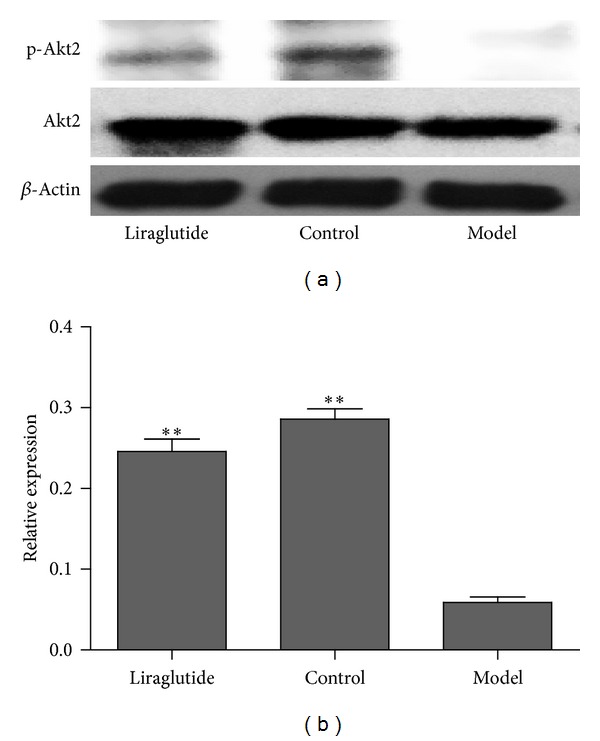
Effects of liraglutide on p-Akt2 and Akt2 expressions in skeletal muscle. (a) Expression in skeletal muscle of p-Akt2 and Akt2 mRNA in normal, model, or liraglutide group. Gene expression was measured by RT-PCR. Relative p-Akt2 and Akt2 mRNA abundance was normalized by *β*-actin. Data were shown as mean ± SD, *P < 0.05, **P < 0.01 versus model group, *n* = 5. (b) Expression of p-Akt2 and Akt2 protein in skeletal muscle from normal, model, or liraglutide group. Lysates from freshly isolated skeletal muscle tissues were measured for p-Akt2 and Akt2 expression by Western blot analysis. Results were shown as mean ± SEM, *P < 0.05, **P < 0.01 versus model group, *n* = 5.

**Figure 7:**
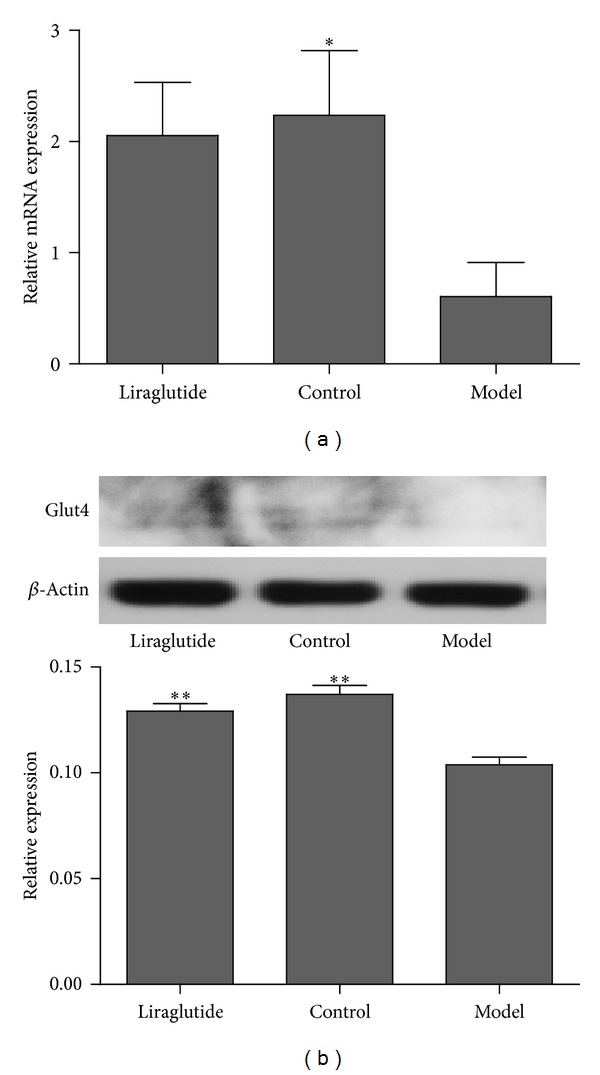
Effects of liraglutide on GLUT4 expression in skeletal muscle. (a) Expression in skeletal muscle of GLUT4 mRNA in normal, model, or liraglutide group. Gene expression was measured by real-time PCR. Relative GLUT4 mRNA abundance was normalized by *β*-actin. Data were shown as mean ± SEM, *P < 0.05, **P < 0.01 versus model group, *n* = 5. (b) Expression of GLUT4 protein in skeletal muscle from normal, model, or liraglutide group. Lysates from freshly isolated skeletal muscle tissues were measured for GLUT4 expression by Western blot analysis. Results were shown as mean ± SEM, *P < 0.05, **P < 0.01 versus model group, *n* = 5.

To evaluate the mechanisms of liraglutide on antidiabetic effect, the major proteins in PTP1B and PI3K/Akt signaling pathway were assessed using Western blot after treatment with liraglutide for 6 weeks. PTP1B in the control and liraglutide groups was downregulated significantly when compared with the model group (*P* < 0.01) (Figure 4(b)). The level of PI3K had significant difference between liraglutide group and the model group (*P* < 0.01) (Figure 5(b)). In addition, p-Akt2/Akt2 in skeletal muscle was significantly increased in liraglutide group, compared with nontreated model group (*P* < 0.01); however, Akt2 protein expression did not change (Figure 6). Moreover, GLUT4 was significantly upregulated in the control and liraglutide groups compared with the model group (*P* < 0.01) (Figure 7(b)).

## 4. Discussion

As a novel GLP-1 analogue, liraglutide had been used to treat diabetes since 2009 and was also reported to have the effect of improving glycemic control in diabetic mice [20], which decreased the occurrence of hyperglycemia. In our previous results, we had demonstrated that liraglutide could increase glycogen level and further improve glycometabolism [14]. However, mechanisms of improving glycometabolism and antidiabetic effect were still unknown. Thus, to further assess these mechanisms of liraglutide in skeletal muscle, we examined PI3K/Akt signaling pathway, a classic pathway which was vital for diabetes study, after He et al. studied diabetic cardiomyopathy [21], Cao et al. studied glucose homeostasis [22], and Zhang et al. discovered the injury of high glucose through the PI3K/Akt pathway [23]. We examined the mRNA expression of PTP1B, PI3K, and GLUT4 in skeletal muscle by real-time PCR to determine whether liraglutide produced antidiabetic effects through these signal molecules. Protein expression of the above molecules and p-Akt2/Akt2 in skeletal muscle was also measured by Western blot.

PTP1B was a major molecule in insulin signal transduction [13, 24] and was reported to negatively regulate insulin transduction by dephosphorylating IR and IRS [25]. Several reports had demonstrated that PI3K was a member of insulin signaling system [26, 27], along with insulin receptors (IR) and insulin receptor substrates (IRS), and was modulated by them. Therefore, the change of the level of PTP1B was associated with the expression of PI3K; that is, PTP1B was able to indirectly regulate the expression of PI3K. This was the first time to assess whether liraglutide could modulate insulin signaling pathway through influencing PTP1B. In this study, administration of liraglutide in diabetic mice significantly decreased the PTP1B expression along with higher PI3K expression compared with the diabetic group treated with equivalent volume of normal saline. Decreased level of PTP1B demonstrated that liraglutide exerted antidiabetic effect in skeletal muscle by reducing the expression of PTP1B. Some other reports also revealed the relationship between PTP1B and diabetes. Stull et al. studied skeletal muscle PTP1B gene expression in human [13]. They discovered that PTP1B was significantly higher in diabetic subjects compared with the subjects without diabetes. In addition, another report with mice of knockoff of PTP1B found an effect of antidiabetes [28]. Swarbrick et al. suggested that PTP1B was the negative regulator of glycometabolism in monkeys [29].

Previously, PI3K, as an important molecule in insulin signaling pathway, had been found to play a vital role in diabetes [30, 31]. And so many people discovered it in their study, which was associated with diabetes. Yang et al. examined the level of PI3K in* db*/*db* mice by Western blot and ELISA, showing that diabetes-induced suppression of PI3K may be related to oxidative stress [32]. In addition, Sato-Miyata et al. determined the expression of PI3K to prove the damage of high level insulin in drosophila [33]. In this study, we also investigated PI3K and found that liraglutide enhanced the expression of PI3K in skeletal muscle tissue significantly, suggesting that liraglutide may be able to improve insulin transduction through the PI3K/Akt signaling pathway. These findings were in accordance with the study in liver tissue of diabetic mice, showing that PI3K was decreased in diabetic rats compared with normal rats [34].

Akt2, as an isoform of Akt, played a central role in insulin signaling and glucose metabolism. It was all known that Akt2 was regulated by the activity of PI3K in skeletal muscle tissue. Significant increase in p-Akt2/Akt2 ratio was observed in normal mice compared with diabetic mice, which proved that Akt2 was a major modulator of diabetes. Previous studies also studied the expression and the activity of Akt2 in diabetic mice. To investigate the effect of garlic on cardiac complications in diabetic rats, Padiya et al. examined the level of p-Akt2/Akt2 ratio by Western blot [35]. Sajan and Farese studied the protein level of Akt2 in hepatocytes of type 2 diabetic humans to prove the importance of protein kinase C-1 in insulin signaling [36]. As shown above, there had been several reports that argued the relationship between diabetes and Akt2. However, few reports studied the effect of Akt2 in liraglutide treated diabetic mice. So, to determine the mechanisms of antidiabetic effect of liraglutide, we investigated the expression of Akt2 in diabetes treated with liraglutide. We found that the p-Akt2/Akt2 ratio had a significant increase in liraglutide treated diabetic mice compared with diabetic mice. This result suggested that liraglutide improved diabetes through regulating the activity of Akt2.

In addition, the increased p-Akt2/Akt2 ratio led to the enrichment of GLUT4. Investigation of the mechanisms of liraglutide to protect against T2DM was examined by studying the levels of molecules in the PI3K/Akt signaling pathway. The data from the real-time PCR and Western blot indicated that GLUT4 was enhanced significantly with liraglutide treatments in comparison to diabetic mice. Our results were in line with the followed reports. Shih et al. discovered GLUT4 in skeletal muscle was greater in medicated groups than diabetic group [37]. Their results revealed the relationship between hypoglycemic effect of CNE and glucose uptake in skeletal muscle. The action of GLUT4 in inflammation was destroyed in diabetes. As the insulin downstream effector, the deficiency of GLUT4 was considered closely related to the function of podocyte in diabetic nephropathy mice [38]. Their data indicated that GLUT4 deficiency could protect mice from diabetic nephropathy.

In spite of the fact that the effect of liraglutide on the quality of mitochondrial ultrastructure had been studied by Schwasinger-Schmidt et al. [39], little had been done to discover the role of liraglutide on the other respect of mitochondria. In our study, we observed that all of the individual mitochondrial area, mitochondrial area/100 *μ*m^2^, and mitochondrial number/10 *μ*m^2^ in skeletal muscle were significantly increased in mice treated with liraglutide when compared with the diabetic mice in a model group. Vacuolated, disarranged, and swollen mitochondria clusters were also improved. It has been known that liraglutide could reduce cardiac lipid accumulation [40]. Our study showed that lipid droplets in skeletal muscle were decreased, similar to the conclusion above. In addition, liraglutide greatly ameliorated atrophy and derangement of myofibrils and sarcomere. From these results, we revealed that liraglutide, a protective drug for T2DM, could improve the damage of mitochondria from the number and area and ameliorate myofibril damage and accordingly suppress oxidative stress.

In conclusion, our data indicated a strong link between liraglutide and PTP1B as evidenced by its effect on diabetes. We found that liraglutide caused downregulation of PTP1B and upregulation of PI3K, p-Akt2/Akt2, and GLUT4 and eventually led to the improvements of myofibrils and mitochondrion in skeletal muscle. Furthermore, GLUT4 played a vital role in glucose uptake through the differential regulation of PTP1B, PI3K, and Akt2. It revealed that liraglutide could improve myofibril and mitochondria injury in skeletal muscle against T2DM via PTP1B and PI3K/Akt2 signaling pathway.
